# Complete Genome Sequence of a Novel Avian Paramyxovirus (APMV-13) Isolated from a Wild Bird in Kazakhstan

**DOI:** 10.1128/genomeA.00167-16

**Published:** 2016-05-19

**Authors:** K. Karamendin, A. Kydyrmanov, A. Seidalina, S. Asanova, M. Sayatov, E. Kasymbekov, E. Khan, K. Daulbayeva, S. M. Harrison, I. M. Carr, S. J. Goodman, K. Zhumatov

**Affiliations:** a Laboratory of Viral Ecology, Institute of Microbiology and Virology, Almaty, Kazakhstan; b School of Medicine, Faculty of Medicine and Health, University of Leeds, St. James’s University Hospital, Leeds, United Kingdom; c School of Biology, University of Leeds, Leeds, United Kingdom

## Abstract

A novel avian paramyxovirus was identified during annual viral surveillance of wild bird populations in Kazakhstan in 2013. The virus was isolated from a white fronted goose (*Anser albifrons*) in northern Kazakhstan. Here, we report the complete genome sequence of the isolate, which we suggest should constitute a novel serotype.

## GENOME ANNOUNCEMENT

Avian paramyxoviruses (APMVs) are negative-stranded RNA viruses belonging to the genus *Avulavirus* in the family *Paramyxoviridae*, and to date 12 serotypes (APMV-1 to APMV-12) have been identified. APMV 1 to 9 were discovered in the 1970s (1) and since then they have been isolated all over the world. The most important pathogen between them is APMV-1, or Newcastle disease virus, which causes high levels of mortality in poultry. Since 2005, three novel APMV serotypes (APMV 10 to 12) have been isolated from wild bird populations (2–4).

During annual screening of 486 samples collected in 2013 from 348 wild birds belonging to 50 species, two APMV serotypes were identified in samples from northern Kazakhstan: APMV-8, which was not one of the serotypes isolated during the 1970s (5), and a second serotype, which could not be identified.

Here we report the complete genome sequence of a further novel APMV isolate, designated APMV-13, which we characterized using a next-generation sequencing approach.

Hemagglutinating agents were isolated from cloacal swabs after inoculation into 10-day-old embryonated chicken eggs. The isolates did not react during hemagglutination inhibition tests using reference sera specific for serotypes APMV-1 to APMV-9, indicating a potential novel serotype.

For whole-genome sequencing, viral RNA was used as a template for further library preparation using TruSeq Stranded Total RNA with Ribo-Zero Gold (Illumina, USA). Paired-end sequencing was performed on an Illumina HiSeq 3000 instrument. Raw sequence data were assembled and analyzed using CLC Assembly Cell software (Qiagen).

A BLAST search confirmed that the isolated virus sequence grouped within the APMV family but did not match any currently known APMV serotype, and it was therefore designated APMV-13/white fronted goose/Northern Kazakhstan/5751/2013.

Phylogenetic studies were conducted by comparing the complete nucleotide sequence of a novel serotype virus with published nucleotide sequences from the GenBank database using the maximum composite likelihood approach with the Tamura-Nei model in MEGA 6.0 software (6).

The whole-genome sequence of APMV-13 has a length of 15,996 nucleotides, with a GC content of 42.7%, and complies with the “rule of six” characteristic for paramyxoviruses (7). Preliminary review of the annotated genome identified the six genes typical for APMVs: 3′-NP-P/V/W-M-F-HN-L-5,′ which encode eight viral proteins, NP (493 amino acids [aa]), P (397 aa), V (241 aa), W (150 aa), M (366 aa), F (545 aa), HN (549 aa), and L (2,199 aa).

Phylogenetic studies showed that APMV-13 forms a clade with serotypes APMV-1, -9, and -12. Inside this cluster, APMV-13 forms a pair with APMV-12. BLAST comparison among APMV complete genome sequences showed that APMV-13 shares a maximum nucleotide sequence identity at 69% with APMV-12/widgeon/Italy/3920 1/2005, which suggests that APMV-13 should be considered a novel serotype. The F-gene of the novel Kazakh APMV isolate was 96% identical to a sequence of APMV/Shimane/67/2000, which to date has been characterized only for the F-gene, but which had been suggested as a candidate for a new serotype, APMV-13 (8).

### Nucleotide sequence accession number. 

The complete sequence of APMV-13/white fronted goose/Northern Kazakhstan/5751/2013 is available at GenBank under the accession no. KU646513.
